# Dietary Assessment and Factors According to Fruits and Vegetables Intake in Korean Elderly People: Analysis of Data from the Korea National Health and Nutrition Examination Survey, 2013–2018

**DOI:** 10.3390/nu12113492

**Published:** 2020-11-13

**Authors:** Yong-Suk Kwon, Yu-Yeong Yang, Younghee Park, Yoo-Kyoung Park, Sohye Kim

**Affiliations:** 1 Food and Nutrition Division, Department of Agrofood Resources, National Institute of Agricultural Sciences, Jeollabuk-do 55365, Korea; selenium2012@korea.kr (Y.-S.K.); eweew32@korea.kr (Y.-Y.Y.); ypark@korea.kr (Y.P.); 2 Department of Medical Nutrition, Graduate School of East-West Medical Science, Kyung Hee University, Yongin 17104, Korea; ypark@khu.ac.kr; 3 Nutrition Care Services, Seoul National University of Bundang Hospital, Seongnam 13620, Korea

**Keywords:** vegetable, fruit, elderly, KNHANES

## Abstract

This study analyzed dietary assessment and factors according to fruits and vegetables intake in Korean elderly people. We enrolled 8336 Korean elderly people aged ≥65 who participated in the dietary intake survey (24-h recall methods) of the 2013–2018 Korea National Health and Nutrition Examination (KNHANES). The intake of fruits and unsalted/non-starchy vegetables was 372.06 g/day. According to age group, the intake in the age group 65–74 years as 422.47 g/day, and the intake in the age group 75 years + was 301.12 g/day. Based on the intake of daily meals and snacks, the intake of fruits and unsalted/non-starchy vegetables was the highest in snack-eating individuals (480.96 g/day). The subjects who consumed more than the World Health Organization (WHO)/World Cancer Research Fund (WCRF)’s plant food intake standards (over 400 g/day of intake of fruits and unsalted/non-starchy vegetables) were 35.47% of the elderly people. These results suggest that it is necessary to develop more fundamental strategies to increase fruits and vegetables intake among elderly people. Furthermore, the study outcomes are expected to provide basic information for developing education programs to improve the dietary life of Korean elderly people.

## 1. Introduction

Owing to the improvement in national income and living standards and the development of health care technology, the number of elderly people is increasing significantly with an extended life expectancy. The trend is accelerating in the direction of a sharp increase in the proportion of the elderly in the world, and aging in the population is progressing in Korea. As of 2015, the number of elderly people aged 65 years and over accounted for approximately 6.62 million people, accounting for 13.1% and as of 2017, 14.3% of the total population. This proportion will continue to increase in the future, and it is estimated that the proportion of the population aged 65 years and over is expected to more than double in 2030 and reach 40.1% by 2060 [1,2]. Accordingly, there is a growing interest in having a healthy old age. It is emphasized that the risk of malnutrition among elderly people is higher than that in other age groups [3,4]. Lifestyle and an unhealthy dietary pattern are the major triggers in the development of such disease. Therefore, it is important to improve their nutrition. 

In particular, the intake of fruits and vegetables among elderly people is an important part of a healthy diet and is related to various positive health outcomes and a reduced risk of chronic diseases such as cardiovascular disease, stroke, and cancer [5,6,7,8]. As fruits and vegetables generally have a low energy density; are rich in vitamins, minerals, dietary fiber, vegetable sterols, flavonoids, and other antioxidants; and contain beneficial substances such as plant sterol [5,6,7,8], they are regarded as important components in healthy dietary guidelines [9,10,11]. The Korean healthy dietary guidelines contain the content “Eat a variety of foods such as grains, vegetables, fruits, milk and dairy products, meat, fish, eggs, and legumes”, to improve problems such as decreased grain intake, lack of fruits and vegetables intake, increased sugar intake from beverages, increased alcohol intake, excessive sodium intake, and insufficient calcium intake. Therefore, to promote good health, an intake of 400 g/day of fruits and vegetables is a global priority and is actively advocated by national and international health organizations [5,6,7,8,12,13]. The third national health promotion plan has been formulated in Korea [14], and it aims to promote the practice of proper nutrition management and a balanced diet and prevent and manage chronic diseases among the Korean population. This plan also suggests that the proportion of the population who consume more than 500 g of fruits and vegetables will increase by 39.3%, to a maximum of 50%, by 2020. However, elderly people’s [15] intake of fruits and vegetables, according to the World Health Organization, is reported to be less than the recommended 400 g, and this amount is insufficient for elderly people.

This study was conducted using the Korea National Health and Nutrition Examination (KNHANES) data for the period 2013–2018, and Korean elderly people 65 years of age or older were classified according to the WHO/WCRF’s fruits and vegetables intake guidelines into two groups: patients with an intake of approximately 400 g or more and those with no intake. We also aimed to compare the consumption of vegetables between unsalted/non-candied fruit intake and unsalted/non-starchy vegetable intake. By conducting the study as mentioned, we examined the factors that influenced the intake of fruits and vegetables in the elderly population, and by synthesizing these results, we intend to provide basic data for the elderly to lead a sustainable and balanced diet.

## 2. Materials and Methods

### 2.1. Study Design

The KNHANES is a nationwide, population-based, cross-sectional study that aims to assess the health and nutrition status of the Korean civilian, non-institutionalized population. The KNHANES was performed by the Korean Ministry of Health and Welfare and a stratified multistage probability design was used with subject selection made from sampling units using household registries. It also provides basic data for framing a health policy such as improving the nutrition of the people, preventing diseases, and developing health promotion programs [16].

The sample survey plots are extracted from the KNHANES, and it was conducted as a year-round survey between January and December [17].

In addition, the KNHANES consists of health interviews, health examinations, and nutritional surveys, among which the nutritional survey aims to understand the food and nutritional intake level and eating habits of Koreans, and the food frequency questionnaire, dietary life, and food intake according to a 24-h recall method [16].

### 2.2. Subjects

**Figure 1:**
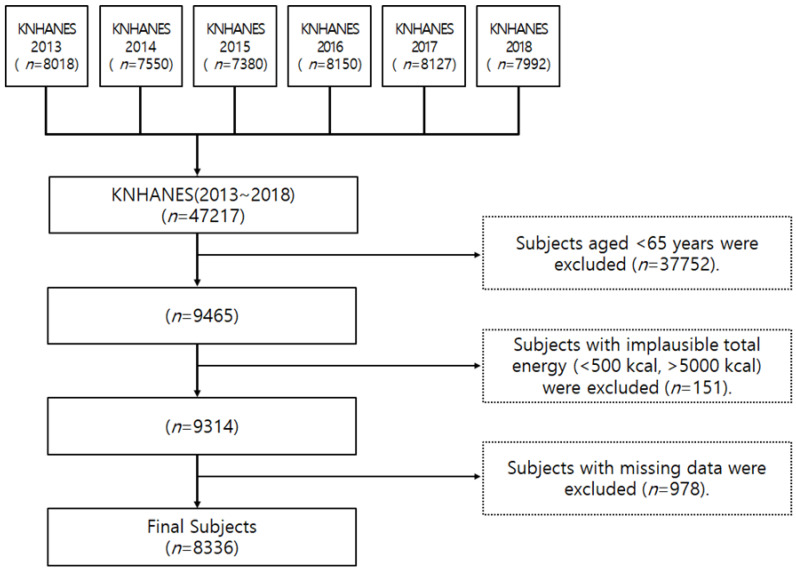
Flowchart of the selection of subjects in this study.

This study selected elderly people aged 65 years and over who participated in the nutritional survey and health interview of the KNHANES 2013–2018. To examine the intake of fruits and vegetables of these subjects, adults who were 65 years of age or older who participated in the 24-h recall method were first selected as subjects (9465 persons). Some of the participants who consumed less than 500 kcal or more than 5000 kcal (151 persons) were excluded. Outlier data of subjects who did not participate in the dietary survey (24 h recall survey) were also excluded (978 persons). A total of 8336 people were included in the study (Figure 1). The KNHANES data used for this study have been approved by the Institutional Review Board of Korea Centers for Disease Control and Prevention (IRB Approval number: 2013-07CON-03-4C, 2013-12EXP-03-5C, and 2018-01-03-PA). KNHANES was exempt from review regarding research ethics based on the Bioethics and Safety Act from 2015 to 2017. However, from 2018, IRB deliberations on the collection of human materials and the provision of raw data to third parties have resumed from 2018 [18].

### 2.3. Fruits and Vegetables Intake

The fruits and vegetables intake survey was classified using the food code variable (variable name: n_fcode) and food intake variable (variable name: nf_intk) of the 24-h recall data based on previous studies [19,20]. Fruit and vegetable juices were excluded from the intake of fruits and vegetables because the exact amount of intake was not known due to the high water content as reported by Kwon et al. [19]. Moreover, starchy vegetables such as potatoes and sweet potatoes were excluded from vegetable intake.

In the first category, vegetable intake was classified into ‘Total vegetable intake’ and ‘Salted vegetables’, such as *kimchi*, *geotjeoli* (a Korean fresh vegetable salad dressed with garlic and chili powder), pickles and other pickles, and ‘Non-starchy/unsalted vegetables’. Intakes for fruits were calculated as total fruit intake of ‘Candied fruits’ and ‘Fresh fruits’. ‘Fresh fruits’ represent all the remaining sorts apart from pickled fruits and jams, which were classified as ‘Candied fruits’. Lastly, ‘Non-starchy/unsalted vegetable’ intake and ‘Fresh fruit’ intake were combined for comparison with the World Cancer Research Fund and World Health Organization’s plant food intake standards [8,12].

In this study, there were 9 types of final classifications for fruits and vegetables intake: ‘Total vegetable’, ‘Unsalted/Non-starchy vegetables’, ‘Salted vegetables’, ‘Vegetable juice’, ‘Total fruits’, ‘Fresh fruits’, ‘Sweetened fruits or candied fruits’, ‘Fruit juice’, and ‘Fruit + Vegetable’ (fresh fruit and non-starchy/unsalted vegetable) [12,19,20].

### 2.4. Covariate

General characteristics of the subjects such as sex, age, marital status, residential area, education level, household income level, occupation, weight status, chewing ability, and stress status were analyzed. Gender, age, occupation, residential area, and household income levels included in the KNHANES raw data were used without modification. However, education level, weight status, and age were modified as follows and used in the study.

First, the education level was classified as under high school graduation (<12 years), high school graduation (12 years), and college degree or more (12 years+). Residential area was classified as city area and rural area, while occupational status was classified into occupation and unemployed. Second, the BMI (Body Mass Index) variable was used for weight status and was based on the Asia-Pacific obesity criteria: if the value is less than 18.5 kg/m^2^, it is underweight; if it is 18.5 kg/m^2^ or more and less than 23.0 kg/m^2^, it is normal; if it is more than 23.0 kg/m^2^ and less than 25.0 kg/m^2^, it is overweight; and if it is more than 25.0 kg/m^2^, then it is obesity (underweight: <18.5 kg/m^2^, normal: ≥18.5 kg/m^2^, <23 kg/m^2^, overweight: ≥23 kg/m^2^, <25 kg/m^2^, obesity: ≥25 kg/m^2^). Moreover, chewing ability was classified into very uncomfortable, uncomfortable, normal, not uncomfortable, and not uncomfortable at all. Lastly, ages were classified into 65–74 years and 75 years or older.

### 2.5. Dietary Behavior

Dietary behavior was analyzed by analyzing ‘breakfast frequency’, ‘meal pattern’, ‘snack’, ‘pattern of cooking place’, ‘food insecurity’, and ‘eating out frequency’. Daily meal types (variable name: N_MEAL) were categorized based on meal time (breakfast, lunch, dinner, or snack), and the subjects who selected ‘4’ (variable value) were classified as ‘ingested’ and ‘not ingested’ otherwise.

The cooking location was based on a variable (variable name: n_mtype) as the presence of eating out, as in the classification used in previous studies [21,22]. ‘Home meal’ represented food prepared at home and lunch box prepared at home, and ‘commercial meal’ represented meals prepared at a restaurant, street stall/store, convenience store, and bakery, instant foods, and others. The meals prepared in school, industry, and senior center, and free-meal service were classified as an ‘institution meal’. The number of instances of eating out (variable name: L_OUT_FQ) was determined by modifying the questionnaire item of the dietary survey. ‘Once a day’ and ‘two or more times a day’ were combined and expressed as ‘one or more times a day’, ‘one to two times a week’, ‘three to four times a week’, and ‘five to six times a week’. ‘One to three times a month’ and ‘less than once a month’ were reclassified to ‘less than once a week’.

Food insecurity is a dietary survey questionnaire that has been included since the 2005 KNHANES, and the question “Which of the following best represents your family’s eating habits over the past year?” was classified based on previous studies [23,24]: an enough food secure category that says “All of our family could eat enough food and a variety of foods as much as they wanted”, and a mildly food insecure category that says “All of our family could eat enough food but couldn’t eat various kinds of food”. Additionally, there was a moderately/severely food insecure category, saying that “there was insufficient food sometimes or often because it was economically difficult”, which was also used for analysis. 

### 2.6. Food Group Classification

To analyze the food intake according to the WCRF/WHO fruits and vegetables intake criteria, the 24-h recall data were used for each individual, and the food group classification was used by reprocessing the KNHANES food group classification 2 (variable name: N_Kindg2) variable. The final classification was obtained by dividing into the following 15 categories: (1) cereal and grain products, (2) potatoes and starches, (3) sugars and sweets, (4) legumes and their products, (5) seeds and nuts, (6) mushrooms, (7) seaweeds, (8) meat and associated products, (9) eggs, (10) fish, (11) milk and dairy products, (12) oils, (13) beverages, (14) seasonings, and (15) others.

### 2.7. Nutrient Intake

Nutrient intake included the measurement of the intake of energy, carbohydrate, fat, protein, mineral, and vitamins over the 24-h period for each subject. However, vitamin A intake was calculated as retinol equivalents (variable name: NF_VA_RE), which is retinol + 1/6 * beta-carotene {(KNHANES VI (2013–2015)}, as well as RAE (Retinol Activity Equivalents, variable name: NF_VA_RAE), which is retinol + 1/12 * beta-carotene, from the first year of KNHANES VII (2016) since the standard of vitamin A evaluation was changed in 2016 [25]. In this study, the RAE variable was used in the same manner as the existing bases for the vitamin A intake of the KNHANES VII (2016–2018) data for an integrated nutrient analysis of all data.

### 2.8. Statistical Analysis

The statistical analyses in this study were conducted by adopting stratification, clustering, and weight variables using SAS (Statistical Analysis System, ver. 9.4, SAS Institute, Cary, NC, USA,) by applying a significance level of *p* < 0.05. Since the KNHANES data are based on multi-stage stratified colony sampling, the analysis was performed considering the stratification variable (Strata: Kstrata), the colony variable cluster: PSU (primary sampling unit), and weight (Wt_ntr).

Data of categorical variables such as general subjects and dietary factors related to the dietary life of the subjects are expressed as frequency (*n*) and weighted percentage (Weighted %) using frequency analysis, and the significance test was tested using the chi-square test. Data of continuous variables such as nutrients and food intake are expressed as mean and standard error using descriptive analysis, and the significance test for these variables was performed using PROC SURVEYREG to calculate the *p*-value. The intake of fruits and vegetables according to gender was adjusted for age and energy intake, and the intake of fruits and vegetables by age was adjusted for gender and energy intake. Other nutrient intakes excluding the intake of fruits and vegetables were analyzed after adjusting for gender, age, and energy intake. Energy intake was adjusted for gender, age, and total food intake. In the analysis of factors that influenced the increase and decrease in the intake of ‘Fresh fruits + Non-starchy/unsalted vegetables’ of 400 g or more per day, which is recommended by the WCRF/WHO, general factors were used as independent variables. Groups with more than 400 g of intake were classified as 1, and those who did not have intake were classified as zero (zero points) and used as dependent variables. These variables were evaluated for odds ratios (ORs) and 95% confidence interval (95%CI) using multivariate logistic regression analysis. At this time, energy intake was used as an adjusted variable.

## 3. Results

### 3.1. Fruits and Vegetables Intake in Elderly People

From 2013 to 2018, the intake of total vegetables, unsalted/non-starchy vegetables, salted vegetables, vegetable juice, total fruits, unsalted/non-candied fruits, candied fruits, fruit juice, and ‘Fruits and vegetables’ (fresh fruit intake + unsalted/non-starchy vegetable intake) in elderly people aged 65 years and over is shown in Table 1. The average total vegetable intake was 312.99 g, of which the intake of unsalted/non-starchy vegetables was 192.27 g, salted vegetables 118.29 g, and vegetable juice 2.43 g. Total fruits intake was 183.69 g, of which the intake of unsalted/non-candied fruits was 179.8 g, candied fruits 0.40 g, and fruit juice 3.49 g. In addition, the intake of fruits and vegetables combined with unsalted/non-starchy vegetables and unsalted/non-candied fruits was 372.06 g. Therefore, 64.53% of subjects who consumed less than 400 g (<400 g), which is the limit recommended by the WHO/WCRF, and 35.47% of those who consumed 400 g or more (≥400 g) were included in the survey.

### 3.2. General Characteristics According to Grouping of Recommended Fruits and Vegetables Intake

Table 2 shows the general characteristics according to the recommended fruits and vegetables (unsalted/non-starchy vegetables + unsalted/non-candied fruits) intake for each group (less than 400 g and more than 400 g).

Men’s average intake was 397.54 g, while women’s average intake was 353.13 g; men had more fruits and vegetables intake, and the rate of the “≥400 g” intake was 52.72% for women and 47.28% for men. In the group aged 75 years or older, the rate of ingesting ‘≥400 g’ was 30.43%, which was significantly lower than that of 69.57% in the group aged 65–74 years. The total intake was 422.47 g in the 65–74 years old group, exceeding the recommended 400 g, and in the group over 75 years old, it was as low as 301.12 g. In the city, the subjects who consumed ≥400 g showed a high rate of 78.78%, and as the education level increased, the intake also was significantly increased to 335.55 g for ‘<12 years’, 462.88 g for ‘12 years’, and 550.37 g for ‘≥12 years’. For household income, the ‘middle-low’, ‘middle high’, and ‘high’ groups excluding the ‘low’ group had an intake of 400.62, 440.42, and 500.17 g, respectively, which exceeded the 400 g intake. Intake in the unemployed group (373.69 g) was lower than that in the employed group (398.74 g). For weight status, in the group excluding ‘underweight’, an intake of ≥400 g appeared to be higher.

In addition, there was a difference in fruits and vegetables intake for each group in chewing ability. The group “very uncomfortable” and ‘uncomfortable’ showed an intake lower than 400 g, whereas the groups ‘normal’, ‘not uncomfortable’, and ‘not uncomfortable at all’ had an intake higher than 400 g. There was also a difference in intake according to the stress levels. In the ‘feel it very much’ group, the average intake was the lowest, at 313.10 g, and the proportion of the subjects with an intake of ‘≥400 g’ was the lowest in terms of the stress levels.

### 3.3. Dietary Behavior According to Grouping of Recommended Fruits and Vegetables Intake

Table 3 shows the differences in dietary behavior according to the recommended intake of fruits and vegetables (unsalted/non-starchy vegetables + unsalted/non-candied fruits) as recommended by the WHO/WCRF in different groups (less than 400 g and more than 400 g).

As for the breakfast frequency, in the ‘five to seven times a week’ group, the rate of ‘≥400 g’ intake was higher, with the highest intake at an average of 375.50 g and the lowest at ‘<1/week’ at an average of 285.27 g. The difference according to the meal pattern was 414.04 g on average in the ‘B + L + D (B: breakfast, L: lunch, D: dinner)’ group, exceeding 400 g, and the rate of ‘≥400 g’ was the largest, but the average in all other groups was less than 400 g. Snack was found to have a high rate of 83.21% for an intake of ‘≥400 g’ in the ’yes’ group, and the average amount was 480.96 g, while in the ‘no’ group, it was low, at 216.09 g. In the pattern of cooking place, the proportion of the ‘≥400 g’ group in ‘H + C (home + commercial location)’ was the highest, at 79.32%, with an average of 452.25 g. ’H + C + I (H: home, C: commercial location, I: institution)’ was the highest with an average of 501.84 g. ‘C + I (commercial location + institution)’ was 369.85 g and we found 344.18 g in ‘only commercial location’, while the average of 191.36 and 119.24 g in ‘only home’ and ‘only institution’ was low, respectively. The difference according to the ‘eating out frequency’ was ‘<1/week’, and the ‘<400 g’ intake rate was 61.37% in the group with a small eating out rate, indicating that they ate the least amount of fruits and vegetables.

### 3.4. Food Intake According to Grouping of Recommended Fruits and Vegetables Intake

Table 4 shows the food intake for each group (less than 400 g and more than 400 g) according to the recommended intake of fruits and vegetables (unsalted/non-starchy vegetables + unsalted/non candied fruits) of the WHO/WCRF.

Compared to the ‘<400 g’ group, ‘total food’, ‘fruits and vegetables’, ‘total vegetables’, ‘fresh vegetables’, ‘total fruits’, ‘seeds and nuts’, ‘mushrooms’, ‘seaweeds’, ‘eggs’, ‘fish’, ‘milk and dairy products’, ‘oils’, ‘beverages’, and ‘seasonings’ were all significant when unadjusted and adjusted for gender, age, and energy. ‘Salted vegetables’, ‘candied fruits’, ‘cereal and grain products’, ‘potatoes and starches’, ‘sugars and sweets’, ‘legumes and their products’, and ‘meat and associated products’ were significant when unadjusted. They were significantly higher when unadjusted, but they were not significant when adjusted for gender, age, and energy. For ‘fresh fruit’, it was not significant when unadjusted but significantly higher when adjusted for gender, age, and energy.

### 3.5. Nutrient Intake According to Grouping of Recommended Fruits and Vegetables Intake

Nutrient intake according to the grouping of recommended fruits and vegetables intake is shown in Table 5. In all nutrients, including energy, the intake of ‘≥400 g’ was significantly higher than that of ‘<400 g’. For the ‘fat’ of the energy contribution ratio and the “protein” of the energy contribution ratio, the intake of ‘≥400 g’ was higher than that of ‘<400 g’, but only for ‘carbohydrate’ of the energy contribution ratio, the intake of ‘≥400 g’ was significantly lower than that of ‘<400 g’.

In the results of a survey divided into two groups, namely ‘65–74 years old’ and ‘≥75 years old’, all nutrient intakes were significantly higher in the ‘≥400 g’ group than in the ‘<400 g’ group. The intake with only ‘carbohydrate’ as the source of energy was significantly lower in the ‘≥400 g’ group than in the ‘<400 g’ group.

### 3.6. Factors Related to Fruits and Vegetables Intake, General Characteristics, and Dietary Behavior

Table 6 shows the results of factors related to the intake of fruits and vegetables according to the WCRF/WHO criteria for the elderly in Korea by age and overall.

Among the total elderly people, the number of people who consumed fruits and vegetables in the <400 g group tended to decrease by approximately 3.4% (OR = 0.966) each time their age increased by one year. In terms of gender, women’s intake of fruits and vegetables of 400 g or more increased by 1.46 times (OR = 1.459) compared to that of men, and the intake in rural areas (rural area) decreased by 21.2% (OR = 0.788) compared to that in urban consumers (city). The intake according to household income level increased by 1.21 times (OR = 1.208) for the low-income group (low) and by approximately 1.49 times (OR = 1.486) for the high-income group. In the case of education level, intake of fruits and vegetables increased by approximately 1.595 times (OR = 1.595) for 12 years (high school) and by 2.38 times (OR = 2.376) for ≥ 12 years (college or higher) based on < 12 years (middle school or lower).

In the snack group, the intake increased by approximately 4.75 times (OR = 4.746) in the ‘yes’ group than in the ‘no’ group, and the proportion of subjects who ate more than 400 g of fruits and vegetables was found to be higher. The daily meal pattern of ‘B + D’, ‘B + L’, ‘L + D’, and all others (when eating only one meal a day) compared to that in the group who consumed ‘B + L + D’ significantly decreased by 33.0–52.6% of fruits and vegetables intake. In the case of the pattern of cooking location, compared to the group of ‘H + C + I’, the ‘only home’ group had an intake that decreased by 55.7% (OR = 0.443), whereas the group that consumed ‘only institution’ had an intake that decreased by 89.6% (OR = 0.106). The ‘H + I’ group had an intake that decreased by 63.9% (OR = 0.361). 

In the case of food insecurity, the intake significantly decreased by approximately 38.8% (OR = 0.612) in the ‘mildly food insecure’ group compared to that in the ‘enough food secure’ group. In the 65–74 years old age group, women’s intake of fruits and vegetables for 400 g or more increased by 1.54 times (OR = 1.544) compared to that of men, and the intake in residential areas increased by 23.2% (OR = 0.768) compared to that of urban consumers. The intake by level of household income increased by approximately 1.40 times (OR = 1.399) for the group above the income level compared to that of the group below the income level.

In terms of education level, the intake of fruits and vegetables increased by approximately 1.49 times (OR = 1.488) for 12 years (high school) and by approximately 2.76 times (OR = 2.762) for ≥ 12 years (college or higher) compared to < 12 years (middle school or lower). In the snack group, the proportion of subjects who ate more than 400 g of fruits and vegetables was found to be approximately 4.55 times (OR = 4.547) higher in the group who ate this amount than in the group who did not. The daily meal pattern of ‘B + D’, ‘B + L’, ‘L + D’, and ‘others’ for fruits and vegetables had significantly decreased by 34.9–72.8% compared to that of the group who consumed ‘B + L + D’.

In the case of the pattern of cooking location, the number of subjects using ‘H + C’ decreased by 35.5% (OR = 0.645) compared to the number of patients who used ‘H + C + I’. In the case of food insecurity, the intake significantly decreased by approximately 43.5% (OR = 0.565) in ‘mildly food insecure’ compared to that of ‘enough food secure’.

The results of the age group over 75 years are as follows: Based on the income level of the ‘low’ group, the income level of the ‘high’ group increased by approximately 1.76 times (OR = 1.763). In terms of education level, the intake of fruits and vegetables increased by approximately 1.83 times (OR = 1.825) for 12 years (high school) and by approximately 1.67 times (OR = 1.673) for ≥ 12 years (college or higher) compared to 12 year (middle school or lower). Snack was found to be approximately 5.60 times (OR = 5.602) higher in ‘yes’ group than in ‘no’ group in subjects who ate more than 400 g of fruits and vegetables. The daily meal pattern decreased by 36.1% (OR = 0.639) in the group eating only ‘L + D’ compared to that in the group ingesting all ‘B + L + D’, and it decreased by 64.7% (OR = 0.353) in the ‘others’ group. In the case of the pattern of cooking location, the number of ‘H + I’ subjects decreased by 83.9% (OR = 0.161) compared to that of subjects who used both ‘H + C + I’.

## 4. Discussion

This study investigated the intake of fruits and vegetables in elderly Koreans aged 65 years and over who were categorized in older groups using the KNHANES data conducted by the Korea Centers for Disease Control and Prevention during 2013–2018. According to the WHO/WCRF [8,13], the intake of fruits and vegetables in elderly people and factors affecting the intake of fruits and vegetables were classified by dividing them into those who consumed more than the recommended limit of 400 g of fruits and vegetables and those who did not. In our study, when the subjects were male, higher age, lived in rural areas, and their household income and education level were low, their intake of fruits and vegetables was low. In addition, there were differences in intake of fruits and vegetables according to snack intake, daily meal pattern, pattern of cooking location, and food insecurity. For vegetables and fruits intake in Koreans in 2012 [24], the average intake of vegetables was 293 g, fruits 174 g, and total vegetables and fruits intake 467 g [24,26]. Vegetables such as kimchi and pickles are a large part of the Korean diet; hence, there is a concern that the intake of sodium increases the risk of high blood pressure, cardiovascular disease, and stomach cancer [27,28,29]. Despite the increase in the intake of vegetables and fruits among Koreans, Korean elderly people have traditionally consumed salted vegetables mainly centered around kimchi and pickles, as well as non-salted vegetables, which raises concerns with regard to the risk of chronic diseases [19].

According to Kwon et al. [19], given that the recommended vegetables and fruits intake was 400 g/day, 25.7% of Korean adults consumed over the recommended amount (satisfy), and 33% of adults do so based on the data from the Centers for Disease Control and Prevention (CDC) in the United States [30]. Therefore, it is fair to conclude that sufficient intake above the recommended amount was low. In another study that examined fruits and vegetables of Korean adults by age group, the proportion of subjects who met the intake criteria over the age of 60 was 18%, about 10% lower than other age groups. Over 28% of those in their 30s, 40s, and 50s all met the inclusion criteria [19].

In our study, the average total intake of fruits and vegetables in people aged over 65 years old was 372.6 g on average, 64.53% of the subjects consumed less than 400 g (<400 g), and subjects who consumed more than 400 g (≥400 g) were found to be 35.47%, and it was found that the proportion of elderly people who consumed fruits and vegetables lower than “400 g” (<400 g) was higher than that of adults who consumed less than “400 g” of fruits and vegetables. The 2002–2003 WHO World Health Survey reported that the prevalence of low fruits and vegetables consumption tends to increase with age and that elderly people did not consume enough fruits compared to the consumption in other age groups [8]. 

In our other results, the rate of intake of 400 g or more (≥400 g) was found to be 30.43% in the 75 years old and over group compared to that of 69.57% in the age group of 65–74 years, and the average intake was 422.47 g in the age group of 65–74 years, which already exceeded the recommended amount of 400 g. However, in the group 75 years and over, the intake was found to be as low as 301.12 g. Among the elderly people 65 years old and over, the higher the age, the lower the intake of fruits and vegetables; hence, it seems that educational data on the nutrition intake guideline for elderly people will be required. 

The higher the income and education level, the higher the intake of fruits and vegetables [19,24,31,32]. The proportion of not only the total subjects but also those in the age groups of “65–74 years” and “75 years” was significantly high compared to that in the “low” intake of fruits and vegetables. Additionally, “enough food security” was also higher in the age group “65–74 years” than “mildly food insecure” and “moderately/severely food insecure”. 

In addition, the consumption of fruits and vegetables was significantly higher in 12 years (high school) and ≥ 12 years (college or higher) than in > 12 years (middle school or lower) with the lowest education level. Depending on household income and education level, there were differences in the intake of fruits and vegetables. The reasons for these differences in the intake level are perceptions of food information and nutritional knowledge [33,34,35,36,37], level of knowledge and education [33,38], and nutritional knowledge that affect food preferences and purchasing behavior [33]. It is explained that nutrition education affects behavior in relation to healthy eating habits of individuals [39].

In our results, the proportion of intake of “400 g” was 52.72% for women and 47.28% for men. The results of our study are consistent with those of studies [40] that have reported a close correlation with higher household incomes and education levels in women [41,42]. This is because women perceive fruits and vegetables as healthy foods compared to men [43], and in women, more awareness and greater self-motivation exist [44].

Depending on the chewing ability, the ratio of “<400 g” was found to be more than “≥400 g” in “very uncomfortable” and “uncomfortable”, whereas “>400 g” was less than “≥400 g” in “normal”, “not uncomfortable”, and “not uncomfortable at all”. The result showed that the ratio of “<400 g” was less than “≥400 g”, and it was found to be a factor affecting the intake of fruits and vegetables according to the chewing ability of elderly people, which is due to the decrease in oral function among elderly people. This finding is in line with that of another study [45,46] that showed that the oral health condition of elderly people is closely related to dietary habits because it interferes with the mastication of food and the digestion and absorption of nutrients, which can cause an imbalance in nutrition.

Therefore, chewing ability must be considered to increase the nutritional status of fruit intake. In terms of fruits intake according to snack consumption, the intake of fruits and vegetables was significantly higher in all subjects in the “yes” than the “no” groups in a study of American elderly people aged 65 years old and over who consumed snacks. The intake of fiber and micronutrients was much higher in the snack group [47,48], and Korean elderly people also reported that snacking increased intake of all micronutrients except vitamin B6 and sodium [46]. It was confirmed that snack consumption not only increases intake of fruits and vegetables but also improves the nutritional status.

Compared to the “group eating three meals a day”, the intake of fruits and vegetables in the “group eating two meals” was significantly lower in “total subjects” and “65–74 years” regardless of any meal pattern. Even in the “over 75-year-old” group, it was significantly lower in “B + D” and “others” (group that ate only one meal), and it was still possible to confirm the importance of having regular meals, which is three times a day. 

In terms of “pattern of cooking place per day”, the consumption of fruits and vegetables was low in “only home” and “H + I”, and “only institution” showed a high intake of fruits and vegetables. In the case of elderly people who eat only at home with less frequency of eating out and little institutional meals, fruits and vegetables may be more vulnerable nutrition for them. Therefore, these older people need more care and attention in their fruits and vegetables intake.

The correlation between the quality of various food intake levels and the quality of fruits and vegetables was proportional. The group with a high consumption of fruits and vegetables showed a high consumption of “cereal and grain products”, “potatoes and starches”, “sugars and sweets”, “legumes and their products”, “meat and associated products”, “seeds and nuts”, “mushrooms”, “seaweeds”, “eggs”, “fish”, “milk and dairy products”, “oils”, “beverages”, and “seasonings”.

The “% Carbohydrate” of the energy contribution ratio was 74.38% for “total subjects”, 73.32% for “65–74 years”, and 75.56% for “75 years of age or older”, which are all above the recommended amount of 55–65% [49], and the carbohydrate ratio was higher in those aged 75 years or older who consumed less fruits and vegetables, and only “carbohydrate” as the source showed a significantly lower intake in the “≥400 g” group than in the “<400 g” group.

As fruits and vegetables are sources of carbohydrates, it is expected that the higher the intake of fruits and vegetables, the higher the carbohydrate ratio; however, the higher the fruits and vegetables intake, the more ideal the energy contribution ratio. From these results, it was found that the better the consumption rate of fruits and vegetables, the better the elderly people consumed different foods and nutrients.

The limitations of this study are as follows. This study examined food intake data involving a single recorded 24-h recall that might not be an accurate representation of the usual intake [50,51]. However, in the 2009 Korea National Health and Nutrition Examination Survey, the difference between a single day 24-h dietary recall and the data obtained for 2 to 10 days was not significantly different (energy: 0.8%, vitamin A: 8.1%, vitamin C: 3.4%) [52]. However, since the subject’s recollection is influenced by short-term memory, it is difficult to measure the exact amount of intake, and it is thought that it is difficult to grasp the level of daily intake due to within-person variation of daily food intake [53]. In order to solve this problem in the future, it is thought that a comparison through a food frequency questionnaire (FFQ) or a long-term follow-up cohort study is necessary.

Despite these limitations, a strength of the study is that it included a large number of participants selected from a nationwide population. In addition, the Korean elderly people’s intake of fruits and vegetables was classified into total vegetable, unsalted/non-starchy vegetables, salted vegetables, vegetable juice, total fruits, unsalted/non-candied fruits, fruit juice, and fruits and vegetables to determine the intake status. In meals and snacks, we conducted a study that compared food intake, nutrients, general characteristics, and dietary behavior. In addition, there is an advantage of conducting research on the population of elderly people aged 65 years old and over, from 65 to 74 years, and 75 years old or older. Most of elderly studies are combined with subjects over 60 or over 65 years old, and there are few papers looking at the age difference in the elderly. However, this study distinguished between the ages of over 75 and under 75 (65–74) in the elderly and found differences between the groups. This could be helpful in nutrition education that differentiates by age group.

## 5. Conclusions

Summarizing the results of the research, to improve the health and nutritional status of elderly people in the future, it is required to focus on the low socioeconomic group, people aged over 75 years, and men rather than women. In addition, it is thought that the elderly who do not eat three meals and those who eat only at home will need measures to increase their intake of fruits and vegetables. To increase the intake of fruits and vegetables, it is necessary to develop sustainable fruit and vegetable menus for the elderly, develop nutrition guidelines and nutrition policies, and provide educational materials through various platforms.

## Figures and Tables

**Table 1:** Intake of fruits and vegetables.

Intake, g/day	Mean	SE ^(1)^
Total vegetables ^(2)^	312.99	3.34
Unsalted/non-starchy vegetables ^(2)^	192.27	2.71
Salted vegetables ^(2)^	118.29	1.89
Vegetable juice ^(2)^	2.43	0.25
Total fruits	183.69	4.07
Unsalted/non-candied fruits	179.80	4.04
Candied fruits	0.40	0.06
Fruit juice	3.49	0.37
Fruits and vegetables ^(2)(3)^	372.06	5.14
Recommended intake of fruits and vegetables ^(4)(5)^ (*n*, weighted %)		
<400 g	5364	64.53
≥400 g	2972	35.47

^(1)^ SE: Standard Error; ^(2)^ Excluding starchy vegetables; ^(^^3)^ fruits and vegetables = unsalted/non-candied fruits intake + unsalted/non-starchy vegetables intake; ^(^^4)^ WCRF (World Cancer Research Fund) and WHO (World Health Organization) recommended fruits and vegetables intake of 400 g/day or more; ^(^^5)^ Intake of fruits and vegetables in this study was defined as intake of unsalted/non-candied fruits and unsalted/non-starchy vegetables.

**Table 2:** General characteristics according to grouping of recommended fruits and vegetables intake.

	<400 g (*n* = 5364)		≥400 g (*n* = 2972)		*p*-Value ^(2)^	Intake (*n* = 8336)		*p*-Value ^(3)^
	*n*	% ^(1)^	*n*	%		Mean	SE	
Gender					<0.0001			<0.0001
Male	2209	40.08	1415	47.28		397.54	6.71	
Female	3155	59.92	1557	52.72		353.13	6.05	
Age					<0.0001			<0.0001
65–74 years	2905	52.36	2104	69.57		422.47	6.61	
≥75 years	2459	47.64	868	30.43		301.12	6.15	
Average (Mean, SE)	73.61	0.09	71.58	0.11	<0.0001 ^(4)^	-		
Marital status					0.7701			0.6591
Unmarried	29	0.55	18	0.61		372.03	5.15	
Married	5331	99.45	2954	99.39		387.18	55.89	
Residential area					<0.0001			<0.0001
City	3701	72.36	2268	78.78		386.60	5.74	
Rural area	1663	27.64	704	21.22		329.29	10.82	
Education level					<0.0001			<0.0001
<12 years	3685	78.67	1623	59.36		335.55 ^c^	5.78	
12 years	684	14.31	599	22.37		462.88 ^b^	12.26	
≥12 years	315	7.02	483	18.27		550.37 ^a^	16.76	
Household income					<0.0001			<0.0001
Low	2873	52.88	1093	36.59		305.10 ^c^	6.04	
Middle-low	1352	25.31	901	29.28		400.62 ^b^	8.83	
Middle-high	685	13.78	541	19.21		440.42 ^b^	12.88	
High	410	8.02	422	14.91		500.17 ^a^	16.62	
Occupation					0.0993			0.0398
Unemployed	3190	69.71	1789	67.59		373.69	6.23	
Employed	1499	30.29	918	32.41		398.74	9.10	
Weight status ^(5)^					0.0027			<0.0001
Underweight	190	3.83	58	2.04		267.56 ^c^	18.34	
Normal	1774	34.29	995	34.93		381.69 ^a^	8.43	
Overweight	1319	25.96	752	26.04		375.12 ^a^	8.57	
Obese	1841	35.92	1051	36.99		375.02 ^a^	7.54	
Chewing ability					<0.0001			<0.0001
Very uncomfortable	741	15.16	273	10.18		307.90 ^c^	11.43	
Uncomfortable	1541	31.74	786	27.24		359.03 ^c^	8.09	
Normal	828	17.05	516	18.68		403.91 ^bc^	11.83	
Not uncomfortable	759	15.50	558	20.05		425.09 ^a^	11.97	
Not uncomfortable at all	964	20.55	644	23.85		400.33 ^b^	10.48	
Stress status					<0.0001			0.0005
Feel it very much	207	4.17	73	2.64		313.10 ^b^	18.42	
Feel somewhat	752	15.80	372	13.96		365.90 ^ab^	11.86	
Feel a little	2311	47.40	1472	52.73		397.38 ^a^	7.10	
Rarely	1564	32.63	859	30.67		369.26 ^ab^	8.10	

^(1)^ Weighted %; ^(2)^ *p*-value calculated using the chi-square test; ^(3)^ *p*-value calculated using the *t*-test or one-way ANOVA; ^(4)^ *p*-value calculated using the *t*-test; ^(5)^ underweight: BMI (Body Mass Index) < 18.5, normal: 18.5 ≤ BMI < 23.0, overweight: 23.0 ≤ BMI < 25.0, obese: BMI ≥ 25.0; ^a–c^ different superscript letters mean significantly different among groups at the α = 0.05 level by Tukey’s multiple range comparison.

**Table 3:** Dietary behavior according to grouping of recommended fruits and vegetables intake.

	<400 g (*n* = 5364)		≥400 g (*n* = 2972)		*p*-Value ^(1)^	Intake		*p*-Value ^(2)^
	*n*	% ^(3)^	*n*	%		Mean	SE	
Breakfast frequency (per week)					0.0234			<0.0001
5–7 times a week	4970	92.43	2824	94.54		375.70 ^a^	5.23	
3–4 times a week	158	2.85	59	2.22		331.24 ^a^	26.24	
1–2 times a week	70	1.28	28	1.05		364.85 ^a^	49.41	
<1/week	157	3.44	56	2.20		285.27 ^b^	22.32	
Meal pattern (per day) ^(4)^					<0.0001			<0.0001
B + L	490	8.86	220	7.79		347.64 ^b^	14.23	
B + D	750	13.86	287	9.70		312.48 ^b^	11.37	
L + D	473	9.42	184	6.68		307.07 ^b^	12.79	
B + L + D	3148	58.11	2188	72.73		414.04 ^a^	6.37	
Others	503	9.76	93	3.10		214.84 ^a^	12.36	
Snack (per day)					<0.0001			<0.0001
No	2954	54.48	503	16.79		216.09	4.95	
Yes	2410	45.52	2469	83.21		480.96	6.38	
Pattern of cooking place (per day) ^(5)^					<0.0001			<0.0001
Only home	2004	36.88	279	9.33		191.36 ^e^	5.80	
Only commercial location ^(6)^	219	4.39	109	3.85		344.18 ^c^	21.23	
H + C	2782	52.04	2376	79.32		452.25 ^b^	6.22	
Only institution ^(7)^	40	0.70	1	0.01		119.24 ^e^	15.02	
H + I	150	2.87	23	0.83		237.31 ^d^	21.36	
C + I	38	0.65	23	0.88		369.85 ^c^	33.32	
H + C + I	131	2.48	161	5.78		501.84 ^a^	24.94	
Food insecurity					<0.0001			<0.0001
Enough food secure	2313	43.87	1605	54.34		410.73 ^a^	7.61	
Mildly food insecure	2611	48.69	1247	42.43		348.45 ^b^	6.56	
Moderately/severely food insecure	404	7.44	107	3.22		245.94 ^c^	12.25	
Eating-out frequency					<0.0001			<0.0001
≥1/day	237	4.61	170	5.93		429.59 ^a^	19.72	
5–6/week	300	5.81	189	6.57		396.95 ^a^	15.91	
3–4/week	333	5.99	277	9.29		463.45 ^a^	16.60	
1–2/week	1221	22.21	869	29.66		425.17 ^a^	9.95	
<1/week	3264	61.37	1462	48.55		360.57 ^b^	7.84	

^(1)^*p*-value calculated using the chi-square test; ^(2)^*p*-value calculated using the *t*-test or one-way ANOVA; ^(3)^ weighted %; ^(4)^ B: breakfast, L: lunch, D: dinner; ^(5)^ H: home, C: commercial location, I: institution; ^(6)^ commercial location meal included Korean, Japanese, Chinese, Western, fast food, instant noodle, instant food, packed meal, convenience food, and bread/cookie; ^(7)^ institution meal included industry, school, religious, elderly, and free; ^a–e^ different superscript letters mean significantly different among groups at the α = 0.05 level by Tukey’s multiple range comparison.

**Table 4:** Food intake according to grouping of recommended fruits and vegetables intake.

Intake, g/day	<400 g		≥400 g		Unadjusted *p*-Value ^(1)^	Adjusted *p*-Value ^(2)^
	Mean	SE	Mean	SE		
Total	*n* = 5364		*n* = 2972			
Total food	988.68	7.82	1745.67	15.48	<0.0001	<0.0001
Cereal and grain products	273.02	2.32	297.94	3.12	<0.0001	0.2663
Potatoes and starches	32.00	1.60	46.12	2.64	<0.0001	0.4972
Sugars and sweets	6.89	0.25	9.65	0.39	<0.0001	0.5680
Legumes and their products	40.86	1.34	48.69	2.25	<0.0001	0.5292
Seeds and nuts	5.71	0.39	11.79	1.07	0.0020	<0.0001
Mushrooms	2.94	0.25	5.33	0.45	<0.0001	0.0285
Seaweeds	22.26	1.46	44.66	3.47	<0.0001	<0.0001
Meat and associated products	49.51	1.71	68.90	2.81	<0.0001	0.1715
Eggs	12.96	0.45	19.61	0.78	<0.0001	0.0473
Fish	46.63	1.61	73.13	2.95	<0.0001	<0.0001
Milk and dairy products	74.01	2.59	120.83	4.98	<0.0001	<0.0001
Oils	3.63	0.10	5.95	0.18	<0.0001	0.0041
Beverages	85.14	3.02	100.95	4.51	0.0111	<0.0001
Seasonings	21.77	0.38	34.86	0.72	<0.0001	<0.0001
Others	1.41	0.26	1.93	0.34	0.2342	0.5762
65–74 years	*n* = 2905		*n* = 2104			
Total food	1075.61	10.51	1816.85	18.50	<0.0001	<0.0001
Cereal and grain products	284.63	3.14	305.52	3.62	<0.0001	<0.0001
Potatoes and starches	35.92	2.29	52.50	3.47	0.0003	0.7623
Sugars and sweets	7.03	0.33	10.26	0.49	<0.0001	0.1836
Legumes and their products	39.81	1.70	50.50	3.01	0.0054	0.9634
Seeds and nuts	6.71	0.53	13.37	1.46	<0.0001	0.0076
Mushrooms	3.44	0.41	5.42	0.48	0.0097	0.2084
Seaweeds	24.61	2.02	46.89	3.82	<0.0001	<0.0001
Meat and associated products	52.90	2.15	73.05	3.53	<0.0001	0.7354
Eggs	15.46	0.65	21.03	0.98	<0.0001	0.2227
Fish	50.35	2.18	75.99	3.57	<0.0001	<0.0001
Milk and dairy products	80.69	3.54	125.54	5.76	<0.0001	0.0002
Oils	4.29	0.15	6.30	0.21	<0.0001	0.1086
Beverages	110.03	4.85	111.37	5.59	0.9279	<0.0001
Seasonings	23.29	0.52	36.19	0.83	<0.0001	<0.0001
Others	1.69	0.35	2.07	0.41	0.3706	0.7694
≥75 years	*n* = 2459		*n* = 868			
Total food	893.14	10.58	1582.98	24.75	<0.0001	<0.0001
Cereal and grain products	260.27	3.11	280.60	5.16	0.0044	<0.0001
Potatoes and starches	27.68	2.06	31.54	3.15	0.3479	0.3057
Sugars and sweets	6.74	0.36	8.25	0.59	0.0224	0.3231
Legumes and their products	42.01	2.12	44.54	2.86	0.2218	0.3146
Seeds and nuts	4.62	0.52	8.17	1.09	0.0065	0.1972
Mushrooms	2.39	0.28	5.10	0.92	0.0088	0.0424
Seaweeds	19.68	2.03	39.57	6.98	0.0204	0.0718
Meat and associated products	45.77	2.66	59.40	4.03	0.0381	0.0911
Eggs	10.21	0.61	16.37	1.19	<0.0001	0.0690
Fish	42.53	2.16	66.57	4.95	0.0002	0.0073
Milk and dairy products	66.67	3.64	110.07	9.12	<0.0001	0.0040
Oils	2.91	0.12	5.16	0.30	<0.0001	0.0033
Beverages	57.78	3.36	77.13	7.52	0.0222	0.4138
Seasonings	20.10	0.54	31.83	1.41	<0.0001	0.0003
Others	1.11	0.33	1.60	0.45	0.5560	0.5150

^(1)^*p*-value calculated using *t*-test. ^(2)^*p*-value calculated using general lineal model (adjusted for gender, age, and energy intake).

**Table 5:** Nutrient intake according to grouping of recommended fruits and vegetables intake.

	<400 g		≥400 g		Unadjusted *p*-Value ^(1)^	Adjusted *p*-Value ^(2)^
	Mean	SE	Mean	SE		
Total	*n* = 5364		*n* = 2972			
Energy (kcal)	1489.49	9.88	1953.77	14.52	<0.0001	<0.0001
Carbohydrate (g)	263.28	1.78	345.65	2.60	<0.0001	<0.0001
Protein (g)	47.45	0.37	66.71	0.68	<0.0001	<0.0001
Fat (g)	22.06	0.29	32.92	0.55	<0.0001	<0.0001
Calcium (mg)	345.95	3.88	549.34	8.09	<0.0001	<0.0001
Phosphorus (mg)	754.95	5.83	1113.24	10.22	<0.0001	<0.0001
Iron (mg)	11.99	0.19	18.18	0.28	<0.0001	<0.0001
Sodium (mg)	2672.00	30.17	3663.48	51.49	<0.0001	<0.0001
Potassium (mg)	2051.24	17.31	3669.93	33.65	<0.0001	<0.0001
Vitamin A (μg RE)	405.54	9.88	763.70	19.18	<0.0001	<0.0001
Carotene (μg)	2254.53	56.76	4318.12	109.99	<0.0001	<0.0001
Retinol (μg)	52.70	2.28	90.26	6.05	<0.0001	<0.0001
Thiamine (mg)	1.28	0.01	1.88	0.02	<0.0001	<0.0001
Riboflavin (mg)	0.88	0.01	1.39	0.02	<0.0001	<0.0001
Niacin (mg)	9.79	0.09	15.11	0.17	<0.0001	<0.0001
Vitamin C (mg)	46.19	0.86	148.68	3.69	<0.0001	<0.0001
Energy contribution (%, SE)						
Carbohydrate	74.38	0.17	71.84	0.23	<0.0001	<0.0001
Protein	12.73	0.06	13.57	0.08	<0.0001	<0.0001
Fat	12.89	0.13	14.59	0.18	<0.0001	0.3377
65–74 years	*n* = 2905		*n* = 2104			
Energy (kcal)	1593.25	13.59	2031.21	17.54	<0.0001	<0.0001
Carbohydrate (g)	275.21	2.42	356.56	3.04	<0.0001	<0.0001
Protein (g)	51.78	0.53	69.78	0.81	<0.0001	<0.0001
Fat (g)	24.91	0.41	34.99	0.65	<0.0001	<0.0001
Calcium (mg)	379.23	5.61	570.96	9.37	<0.0001	<0.0001
Phosphorus (mg)	825.61	8.02	1162.84	11.99	<0.0001	<0.0001
Iron (mg)	12.82	0.17	18.75	0.37	<0.0001	<0.0001
Sodium (mg)	2886.34	40.91	3802.07	63.36	<0.0001	<0.0001
Potassium (mg)	2229.11	22.99	3821.27	41.43	<0.0001	<0.0001
Vitamin A (μg RE)	441.75	12.32	786.78	23.02	<0.0001	<0.0001
Carotene (μg)	2434.66	72.36	4414.70	129.11	<0.0001	<0.0001
Retinol (μg)	59.67	3.01	98.41	8.62	<0.0001	<0.0001
Thiamine (mg)	1.39	0.02	1.95	0.02	<0.0001	<0.0001
Riboflavin (mg)	0.97	0.01	1.45	0.02	<0.0001	<0.0001
Niacin (mg)	10.76	0.12	15.82	0.21	<0.0001	<0.0001
Vitamin C (mg)	49.70	1.11	154.95	4.21	<0.0001	<0.0001
Energy contribution (%, SE)						
Carbohydrate	73.32	0.21	71.33	0.26	<0.0001	<0.0001
Protein	13.01	0.08	13.68	0.09	<0.0001	<0.0001
Fat	13.67	0.17	14.98	0.21	<0.0001	0.3620
≥75 years	*n* = 2459		*n* = 868			
Energy (kcal)	1375.45	12.84	1776.76	22.05	<0.0001	0.0006
Carbohydrate (g)	250.17	2.40	320.73	4.16	<0.0001	<0.0001
Protein (g)	42.70	0.48	59.69	1.06	<0.0001	<0.0001
Fat (g)	18.93	0.37	28.17	0.84	<0.0001	<0.0001
Calcium (mg)	309.38	4.73	499.94	12.65	<0.0001	<0.0001
Phosphorus (mg)	677.30	7.18	999.85	15.83	<0.0001	<0.0001
Iron (mg)	11.09	0.33	16.87	0.36	<0.0001	<0.0001
Sodium (mg)	2436.42	43.54	3346.67	83.64	<0.0001	<0.0001
Potassium (mg)	1855.75	21.80	3324.00	49.97	<0.0001	<0.0001
Vitamin A (μg RE)	365.73	13.73	710.93	33.04	<0.0001	<0.0001
Carotene (μg)	2056.55	77.10	4097.34	195.29	<0.0001	<0.0001
Retinol (μg)	45.04	3.50	71.63	5.85	0.0007	<0.0001
Thiamine (mg)	1.17	0.01	1.73	0.03	<0.0001	<0.0001
Riboflavin (mg)	0.78	0.01	1.25	0.03	<0.0001	<0.0001
Niacin (mg)	8.73	0.12	13.48	0.25	<0.0001	<0.0001
Vitamin C (mg)	42.34	1.13	134.36	5.04	<0.0001	<0.0001
Energy contribution (%, SE)						
Carbohydrate	75.56	0.24	73.01	0.42	<0.0001	<0.0001
Protein	12.42	0.09	13.31	0.16	<0.0001	<0.0001
Fat	12.03	0.18	13.68	0.32	<0.0001	0.8242

^(1)^*p*-value calculated using *t*-test; ^(2)^*p*-value calculated using general lineal model (energy intake was adjusted for gender, age, and food intake; other nutrients were adjusted for gender, age, and energy intake.

**Table 6:** Factors related to fruits and vegetables intake, general characteristics, and dietary behavior ^(1)(2)^.

	Total (*n* = 8336)	65–74 years (*n* = 5009)	75 years+ (*n* = 3327)
Age	0.966 (0.951–0.981) ^(3)^ *	0.971 (0.942–1.001)	0.955 (0.898–1.015)
Gender (Ref. = ‘Male’)			
Female	1.459 (1.259–1.692) *	1.544 (1.284–1.856) *	1.219 (0.928–1.601)
Region (Ref. = ‘City’)			
Rural area	0.788 (0.652–0.952) *	0.768 (0.611–0.965) *	0.829 (0.615–1.118)
Marital status (Ref. = ‘Unmarried’)			
Married	1.034 (0.299–3.580)	1.741 (0.508–5.973)	0.288 (0.060–1.378)
Household income (Ref. = ‘Low’)			
Middle-low	1.208 (1.015–1.439) *	1.188 (0.972–1.453)	1.269 (0.918–1.754)
Middle-high	1.151 (0.919–1.442)	1.130 (0.877–1.456)	1.259 (0.825–1.921)
High	1.486 (1.111–1.988) *	1.399 (1.010–1.939) *	1.763 (1.054–2.949) *
Education level (Ref. = ‘<12 years’)			
12 years	1.595 (1.326–1.918) *	1.488 (1.187–1.867) *	1.825 (1.314–2.535) *
≥12 years	2.376 (1.849–3.054) *	2.762 (2.034–3.751) *	1.673 (1.065–2.628) *
Obese status (Ref. = ‘Normal’)			
Underweight	0.835 (0.531–1.311)	0.893 (0.472–1.689)	0.787 (0.402–1.537)
Overweight	0.938 (0.791–1.111)	0.895 (0.724–1.105)	1.048 (0.769–1.430)
Obesity	0.998 (0.854–1.166)	0.931 (0.768–1.129)	1.165 (0.886–1.533)
Stress status (Ref. = ‘Feel it very much’)			
Feel somewhat	1.299 (0.878–1.922)	1.255 (0.805–1.958)	1.525 (0.625–3.719)
Feel a little	1.287 (0.895–1.852)	1.206 (0.803–1.814)	1.545 (0.674–3.541)
Rarely	1.292 (0.888–1.882)	1.256 (0.810–1.948)	1.471 (0.639–3.389)
Chewing ability (Ref. = ‘Very uncomfortable’)			
Uncomfortable	0.784 (0.617–1.001)	0.918 (0.671–1.256)	0.595 (0.419–0.847)
Normal	0.900 (0.694–1.167)	0.947 (0.676–1.327)	0.850 (0.574–1.260)
Comfortable	0.949 (0.726–1.241)	1.017 (0.723–1.431)	0.881 (0.584–1.330)
Very comfortable	0.977 (0.752–1.268)	1.046 (0.742–1.476)	0.901 (0.620–1.310)
Breakfast frequency (Ref. = ‘<1/week’)			
5–7 times a week	0.888 (0.589–1.338)	0.898 (0.575–1.402)	0.957 (0.376–2.437)
3–4 times a week	1.032 (0.621–1.715)	1.436 (0.798–2.585)	0.266 (0.059–1.208)
1–2 times a week	1.424 (0.880–2.302)	1.529 (0.841–2.782)	1.361 (0.614–3.018)
Snack per day (Ref. = ‘No’)			
Yes	4.746 (3.909–5.762) *	4.547 (3.617–5.717) *	5.602 (3.892–8.063) *
Meal pattern per day (Ref. = ‘B + L + D’)			
B + L	0.670 (0.521–0.861) *	0.586 (0.434–0.793) *	0.882 (0.560–1.387)
B + D	0.646 (0.520–0.803) *	0.651 (0.502–0.845) *	0.639 (0.454–0.901) *
L + D	0.474 (0.350–0.643) *	0.394 (0.278–0.558) *	0.695 (0.406–1.191)
Others	0.311 (0.226–0.428) *	0.272 (0.181–0.410) *	0.353 (0.204–0.611) *
Pattern of cooking place per day (Ref. = ‘H + C + I’)			
Only home	0.443 (0.289–0.679) *	0.377 (0.228–0.624)	0.586 (0.277–1.239)
Only commercial	0.907 (0.539–1.526)	0.736 (0.386–1.402)	1.387 (0.572–3.364)
H + C	0.728 (0.511–1.036)	0.645 (0.423–0.983) *	0.855 (0.469–1.559)
Only institution	0.106 (0.012–0.946) *	0.141 (0.013–1.471)	-
H + I	0.361 (0.164–0.796) *	0.434 (0.167–1.133)	0.161 (0.040–0.645) *
C + I	1.040 (0.497–2.174)	0.810 (0.334–1.965)	1.389 (0.377–5.114)
Food insecurity (Ref. = ‘Enough food secure’)			
Mildly food insecure	0.612 (0.433–0.865) *	0.565 (0.376–0.849) *	0.691 (0.404–1.182)
Moderately/Severely food insecure	0.755 (0.535–1.066)	0.732 (0.489–1.096)	0.792 (0.463–1.354)
Eating out frequency (Ref. = ‘1/day or more’)			
5–6/week	1.059 (0.692–1.621)	1.038 (0.636–1.695)	0.974 (0.419–2.266)
3–4/week	1.424 (0.974–2.082)	1.480 (0.951–2.305)	1.154 (0.543–2.453)
1–2/week	1.355 (0.972–1.890)	1.421 (0.966–2.089)	1.034 (0.512–2.088)
1–3/month	1.267 (0.914–1.758)	1.225 (0.839–1.789)	1.143 (0.568–2.301)
Rarely	1.196 (0.838–1.708)	1.341 (0.872–2.062)	0.874 (0.422–1.809)

^(1)^ Adjusted for energy intake; ^(2)^ independent variable: ≥400 g (recommended fruits and vegetables intake); ^(3)^ odds ratio (95% confidence interval), * *p* < 0.05.
